# Computerised Dynamic Posturography in Premanifest and Manifest individuals with Huntington’s Disease

**DOI:** 10.1038/s41598-018-32924-y

**Published:** 2018-10-02

**Authors:** Alvaro Reyes, Danielle Salomonczyk, Wei-Peng Teo, Luis D. Medina, Danielle Bartlett, Eva Pirogovsky-Turk, Pauline Zaenker, Jody Corey Bloom, Roger W. Simmons, Mel Ziman, Paul E. Gilbert, Travis Cruickshank

**Affiliations:** 1 Facultad de Ciencias de la Rehabilitacion, Universidad Andres Bello, Santiago, Chile; 2 Department of Psychology, San Diego State University, San Diego, California USA; 3 Institute for Physical Activity and Nutrition (IPAN), Deakin University, Geelong, Australia; 4 Department of Psychology, University of Houston, Houston, Texas USA; 5 School of Medical and Health Sciences, Edith Cowan University, Perth, Australia; 6 Exercise Medicine Research Institute, Edith Cowan University, Perth, Australia; 7 Department of Psychiatry, University of California San Diego, La Jolla, California USA; 8 Department of Neurosciences, University of California San Diego, La Jolla, California USA; 9 School of Exercise and Nutritional Sciences, San Diego State University, San Diego, California USA; 10 School of Biomedical Science, University Western Australia, Perth, Australia; 11 San Diego State University/University of California San Diego Joint Doctoral Program in Clinical Psychology, San Diego/La Jolla, California, USA; 12 Perron Institute for Neurological and Translational Science, Perth, Australia

## Abstract

Evidence from small-scale studies indicates that impairments in postural stability are an early and disabling feature of Huntington’s disease (HD) and may be a useful clinical endpoint for disease modifying trials. Larger studies are needed to confirm these preliminary findings and the suitability of postural stability outcomes as clinical endpoints. Static and dynamic postural stability were evaluated in 54 premanifest HD, 36 manifest HD and 45 healthy individuals using the Sensory Organization Test (SOT) and Limits of Stability (LOS) test. Manifest HD displayed significantly lower scores on all SOT conditions and on the SOT composite score and had more falls than healthy and premanifest HD (p < 0.05). Premanifest and manifest HD demonstrated significantly lower endpoint excursion (p < 0.001), maximum excursion (p ≤ 0.001), and directional control (p ≤ 0.004) values than healthy individuals on the LOS test. Deficits in LOS were found to manifest on the left side of premanifest HD. Significant but low associations were observed between UHDRS-TMS, disease burden score, diagnostic confidence level, SOT conditions and SOT composite score. We confirm here that individuals with premanifest and manifest HD display significant impairments in static and dynamic postural stability. Dynamic posturography assessments should be considered as clinical endpoints for future disease modifying trials.

## Introduction

Impairments in postural stability are a disabling feature of Huntington’s disease (HD) that worsen as the disease progresses and contribute to a deterioration in physical function and an increased risk of falls and fall-related injuries^1–4^. Sensitive assessment of postural stability changes is therefore crucial for preventing falls and monitoring clinical progression of HD.

Studies examining changes in postural stability in individuals with HD have typically used clinical tests and scales such as the Timed Up and Go Test, Functional Reach Test, Tinetti Mobility Test, mini Balance Evaluation System Test, Berg Balance Scale and Activities-specific Balance Confidence Scale^1,2,5–7^. These studies have reported an increased risk of falls, reduced balance confidence, a widened base of support and impairments in postural transitioning and single- and double-legged stance in individuals with manifest HD^1,6,7^. While useful in clinical settings, these measures are unable to detect subtle changes in postural stability and are prone to ceiling and floor effects and rater bias, which limits their usefulness as a clinical endpoint in disease modifying drug trials. These measures also provide little insight into the pathological mechanisms underpinning changes in postural stability in individuals with HD^1–4,8,9^.

Quantitative posturography overcomes many of these limitations, providing a more reliable and valid measure of postural stability in individuals with HD^5,10^. Quantitative posturography assessments are normally undertaken using force plate and computerized dynamic posturography apparatuses. Studies using force plates to examine static and dynamic postural stability have reported significant alterations in centre of mass and pressure and reduced limits of stability in individuals with premanifest and manifest HD^3,8,11^. While informative, these studies have provided little insight into the motor and sensory mechanisms contributing to static and dynamic postural stability impairments in individuals with HD^5^.

Several small-scale studies have examined static and dynamic postural stability and the sensory and motor mechanisms contributing to postural instability in individuals with HD. These studies have found significant impairments in limits of stability, rapid postural adjustments and static and dynamic stability in response to somatosensory, visual and vestibular perturbations in individuals with premanifest and manifest HD^4,8,12^. These findings indicate that postural instability is one of the earliest clinical features of HD and may be useful as a clinical endpoint for upcoming disease modifying drug and rehabilitation trials. However, prior to routine utilization in disease modifying trials, validation of computerized dynamic posturography measures in larger, well-characterized cohorts of individuals with premanifest and manifest HD is required^4,8,12^.

The primary purpose of this study was to evaluate static and dynamic postural stability using computerised dynamic posturography in a large, well-characterized cohort of premanifest HD, manifest HD and healthy age- and gender-matched individuals. This study also sought to examine associations between computerised dynamic posturography and clinical disease measures.

## Methods

### Participants

Ninety individuals with HD (54 premanifest, 36 manifest) and 45 age-and gender-matched healthy controls were recruited for the study. Individuals with HD were recruited from the North Metropolitan Area Mental Health Service (Perth, Western Australia), Deakin University (Melbourne, Victoria), San Diego State University, University of California San Diego and through newspaper advertisement. Characteristics of healthy controls and individuals with premanifest and manifest HD (study groups) are detailed in Table 1. Inclusion criteria were: HD individuals with a CAG repeat ≥39 and the ability to understand and respond to verbal and written instructions given in the study. Exclusion criteria were: no concomitant neurological, cardiovascular, metabolic or vestibular conditions and no recent drug or alcohol consumption. All HD individuals were classified as premanifest or manifest according to the diagnostic confidence level criteria of the Unified Huntington’s Disease Rating Scale-Total Motor Score (UHDRS-TMS)^13,14^. Disease burden score was calculated using the method described by Penney *et al*.^15^. An index to estimate proximity to diagnosis at study entry was obtained using the CAP score. CAP score was calculated by multiplying the age at study entry by a scaling of the CAG repeat length as follows: CAP_S_ = (Age × (CAG-33.66))/432.3326. CAP scores <1, 1 and >1 indicate a 5-year diagnosis probability of <0.5, 0.5 and >0.5, respectively^16^.

**Table 1:** Characteristics of participants in the study (values are mean, standard deviation and p-values).

Clinical Outcomes	Healthy (n = 45)	Premanifest HD (n = 55)	Manifest HD (n = 32)	p-values		
				Healthy versus premanifest HD	Healthy versus manifest HD	Premanifest versus manifest HD
Age (years)	43.96 (10.48)	45.20 (11.99)	49.21 (9.73)	0.902	0.173	0.321
Height (cm)	168.04 (8.00)	168.61 (9.60)	168.37 (7.99)	0.966	0.991	0.995
Weight (Kg)	73.27 (13.55)	75.32 (14.53)	74.88 (13.12)	0.845	0.921	0.994
BMI	25.70 (3.12)	26.08 (3.37)	26.15 (3.04)	0.895	0.884	0.997
Handedness left/right*	2/43	3/52	1/31	0.408	0.384	0.290
CAG repeats	—	42.442 (2.65)	45.18 (3.25)	—	—	<0.001
UHDRS-TMS	—	3.59 (6.03)	30.21 (12.32)	—	—	<0.001
Disease Burden Score	—	280.30 (76.40)	455.42 (112.26)	—	—	<0.001
CAP score	—	0.83 (0.19)	1.26 (0.26)	—	—	<0.001
Diagnostic Confidence Level	—	0.44 (0.68)	4.00 (0.00)	—	—	—

BMI: body mass index, CAG: cytosine-adenine-guanosine, UHDRS-TMS: Unified Huntington’s Disease Rating Scale-Total Motor Score, CAP score: CAG-Age Product Scaled score. *p-values of handedness are based on a two-sample proportion test.

### Study approval, registration, and participants consent

This study was approved by the North Metropolitan Area Mental Health Service (NMAMHS), Edith Cowan University, Deakin University, San Diego State University and University of California San Diego Human Research Ethics Committees. Written informed consent was provided by all participants. All experiments were performed in accordance with relevant guidelines and regulations.

### Procedures

Postural stability was assessed using the Sensory Organization Test (SOT) and Limits of Stability (LOS) test with the Neurocom Smart Balance Master (Natus Medical Incorporated, USA). SOT enables examination of somatosensory, visual and vestibular systems during static and dynamic postural conditions. LOS enables examination of postural stability while participants shift their weight to different directions within their base of support. All participants were familiarized with SOT and LOS tests prior to their administration.

### Measurements

#### Sensory Organization Test (SOT)

The SOT comprises six different sensory conditions: (1) eyes open, fixed support and surroundings (static posturography), (2) eyes closed, fixed support and surroundings, (3) eyes open, fixed support, moving surroundings, (4) eyes open, unstable support, fixed surroundings, (5) eyes closed, unstable support, fixed surroundings, and (6) eyes open, unstable support and moving surroundings. Individuals were required to undertake three twenty-second trials for each sensory condition. For each trial, participants were instructed to stand upright with their arms crossed against their chest. Postural stability performance on each trial was expressed as an equilibrium score, which is calculated by computing the difference between each participant’s sway of the centre of gravity (COG) and a theoretical maximum anterior-posterior sway of 12.5°. When a participant’s COG has minimal or no sway, the difference with the theoretical maximum sway is 12.5°. Values are expressed as a percentage of the theoretical maximum angle of sway, therefore a score of 100 indicates good stability and no movement of the COG. When a participant’s COG moves beyond the limit of stability or the participant has a fall they receive a score of zero. The average of the three trials of each SOT condition was calculated and used for further analysis. A weighted composite score was also generated for each participant by averaging the equilibrium scores for SOT 1 and 2, adding this average to the scores for each trial of tests 3–6 and dividing the sum by the total number of trials. Sensory ratios were also computed to examine specific somatosensory, visual and/or vestibular system deficits. SOMATOSENSORY (SOM) was defined as the participant’s ability to rely on the somatosensory system to maintain postural stability (condition 2/condition 1), VISUAL (VIS) was defined as the participant’s ability to rely on visual system to maintain postural stability (condition 4/condition 1), VESTIBULAR (VEST) was defined as the participant’s ability to rely on vestibular system to maintain postural stability (condition 5/condition 1) and PREF was the extent on which participants rely on visual information to maintain postural stability even when visual information is incorrect (condition 3 + condition 6)/(condition 2 + condition 5).

#### Limits of Stability Test (LOS) test

**Figure 1:**
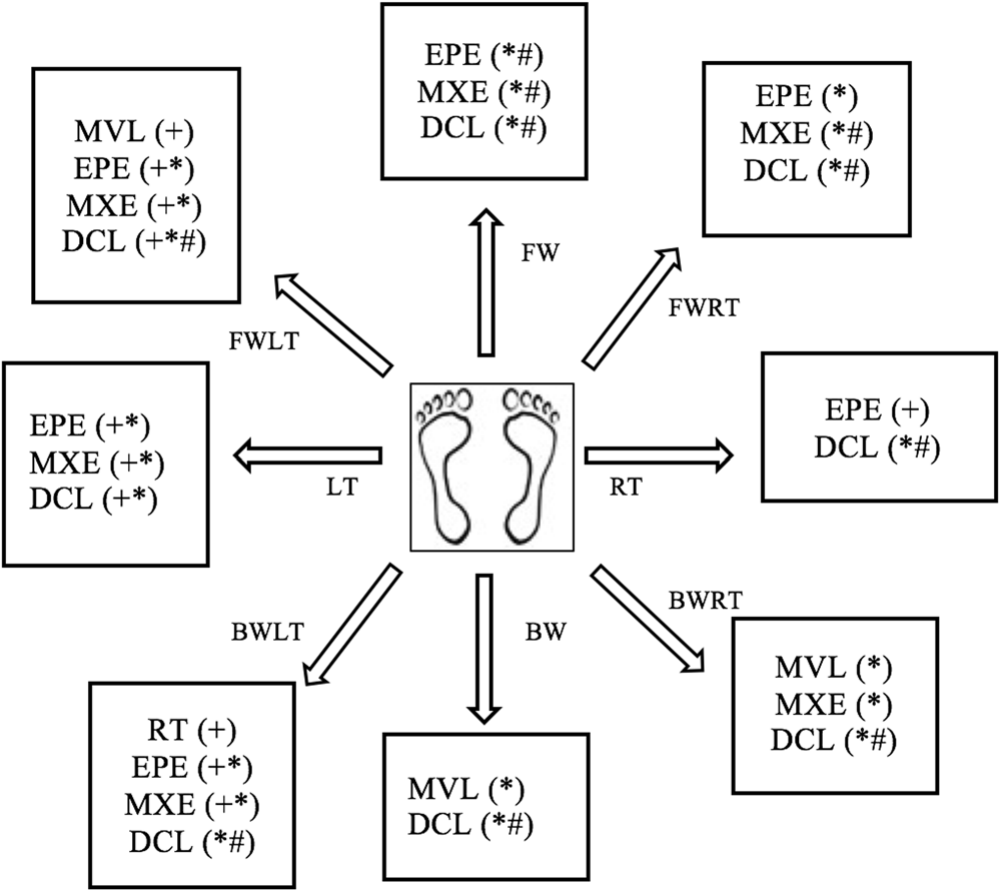
^♦^Schematic representation of specific direction impairments on the limits of stability test. Forward (FW), Forward-Right (FWRT), Right (RT), Backward-Right (BWRT), Backward (BW), Backward-Left (BWLT), Left (LT) and Forward-Left (FWLT). (^+^) significant between control and premanifest HD, (*) significant between control and manifest HD, (^#^) significant between premanifest HD and manifest HD. ^♦^Adapted from Ganesan M, Kanekar N, Aruin AS. Direction-specific impairments of limits of stability in individuals with multiple sclerosis. Annals of physical and rehabilitation medicine. 2015 Jun 1;58(3):145–50.

The LOS test was used to examine dynamic postural stability. Participants were asked to stand comfortably on the force plates and focus on a computer screen placed at eye level to provide visual feedback. Participants were instructed to transfer their weight as quickly and accurately as possible toward one of eight targets displayed on the screen without moving their feet. Targets are located in forward (FW), forward-right (FWRT), right (RT), backward-right (BWRT), backward (BW), backward-left (BWLT), left (LT) and forward-left (FWLT) directions throughout the test. The mean of the eight trials completed for each target was calculated for five LOS dependent variables: reaction time (RT), movement velocity (MV), endpoint excursion (EE), maximum excursion (ME) and directional control (DC). RT represents the time between the presentation of the visual signal to move and the beginning of the participant’s movement, MV is the average speed (degrees/seconds) of the COG in a specific direction, EE is the distance travelled by the COG in the first movement, ME is the farthest distance travelled by the COG and DC is the difference between the amount of movement in the intended direction and the amount of movement the COG deviated form from a straight line. For RT, a higher score represents a poorer performance, while for MV, EE, ME and DC, a higher score represents a better performance. RT and MV are expressed as absolute values and EE, ME and DC are expressed as a percentage of maximum theoretical LOS values as determined by Nashner *et al*. Balance Manager® Systems (2011)^17^. Results are presented using the average of the 8 directions (targets) for each variable (Table 2) and separated by direction (Fig. 1).

**Table 2:** Mean (standard deviation), and p-values of dynamic posturography variables for all groups.

Postural Stability Outcomes	Healthy (n = 45)	Premanifest HD (n = 55)	Manifest HD (n = 32)	p-values		
				Healthy versus premanifest HD	Healthy versus manifest HD	Premanifest versus manifest HD
**Sensory Organization Test**						
Condition 1	94.71 (2.08)	93.15 (2.52)	82.59 (8.44)	1.000	0.004	0.023
Condition 2	92.03 (2.40)	90.53 (4.11)	79.28 (7.72)	1.000	0.002	0.008
Condition 3	92.11 (2.90)	88.17 (6.94)	70.67 (17.92)	1.000	<0.001	<0.001
Condition 4	85.67 (7.92)	84.19 (9.78)	55.19 (23.12)	1.000	<0.001	<0.001
Condition 5	67.74 (9.66)	63.20 (18.86)	28.62 (23.29)	1.000	<0.001	<0.001
Condition 6	68.95 (11.50)	62.95 (20.32)	27.42 (23.54)	1.000	<0.001	<0.001
Composite Score	81.77 (5.12)	78.58 (9.99)	50.80 (16.77)	0.464	<0.001	<0.001
Falls	0.044 (0.20)	0.35 (1.26)	2.80 (3.12)	1.000	<0.001	<0.001
SOM score	0.971 (0.01)	0.971 (0.03)	0.962 (0.06)	1.000	1.000	1.000
VIS score	0.904 (0.08)	0.903 (0.10)	0.654 (0.25)	1.000	<0.001	<0.001
VEST score	0.714 (0.09)	0.676 (0.19)	0.333 (0.26)	0.996	<0.001	<0.001
PREF score	1.00 (0.07)	0.984 (0.11)	0.898 (0.24)	1.000	0.004	0.029
**Limits of Stability Test**						
Reaction time (ms)	1.13 (1.71)	0.79 (0.23)	0.82 (0.18)	0.406	1.000	1.000
Movement Velocity (deg/s)	3.84 (1.30)	3.91 (1.35)	4.83 (1.10)	1.000	0.056	0.078
End Point Excursion (%)	71.53 (10.80)	61.60 (13.96)	53.87 (10.15)	<0.001	<0.001	0.138
Maximum Excursion (%)	89.22 (6.66)	81.88 (11.96)	73.27 (10.72)	0.001	<0.001	0.019
Directional Control (%)	79.27 (6.06)	72.01 (12.55)	43.82 (15.27)	0.004	<0.001	<0.001

SOM: somatosensory (condition 2/condition 1), VIS: visual (condition 4/condition 1), VEST: vestibular (condition 5/condition 1), PREF: (condition 3+ condition 6)/(condition 2+ condition 5).

### Statistical Analyses

Descriptive data are presented as mean and standard deviation. Normality assumptions were tested using a Shapiro-Wilk test. A mixed-factor nested ANOVA was used to compare differences in the six SOT conditions between healthy controls and individuals with premanifest and manifest HD. Significances were adjusted for multiple comparisons using a Bonferroni method after nested analysis of variance (ANOVA). A one-way analysis of variance (ANOVA) was used to compare differences in SOT composite score, falls, SOT sensory ratios and LOS measures between healthy controls and individuals with premanifest and manifest HD. A Bonferroni multiple comparison test was used after ANOVA. All analyses were controlled by study site and the results revealed that study site did not appear to represent a confounding factor. The number of participants recruited and assessed at each study site is provided in Supplementary Table 1. To assess the test-retest reliability of SOT measures, intraclass correlation coefficient (ICC) values were calculated using trial data for each condition. Spearman correlation coefficients were calculated to examine associations between SOT, LOS and UHDRS-TMS, total functional capacity, disease burden score, CAP score, CAG repeat and diagnostic confidence level for premanifest and manifest HD participants. Results of the correlations are presented in scatterplots in Supplementary Fig. 1. Statistical significance was set at p ≤ 0.05. Statistical analyses were performed using STATA version 15.1.

## Results

### Reliability of SOT test

The results of the test-retest reliability of the SOT are presented in Supplementary Table 2. Reliability appeared to be higher for individuals with manifest and premanifest HD than healthy controls. ICC values ranged from 0.27 to 0.62 in healthy controls, from 0.42 to 0.76 in premanifest and from 0.56 to 0.78 in manifest HD.

### SOT test

A two-factor nested ANOVA revealed a significant effect for SOT conditions (F [5;809] = 214.15, p < 0.001), a significant group effect (F [2;809] = 281.22, p < 0.001), and a significant (group × condition) interaction effect (F [10;809] = 11.38 p < 0.001). One-way ANOVA revealed significant differences between groups on the SOT composite score (F [2;132] = 92.64, p < 0.001), falls (F [2;132] = 27.75, p < 0.001), VIS ratio (F [2;132] = 35.12, p < 0.001), VEST ratio (F [2;132] = 45.84, p < 0.001), and PREF ratio (F [2;132] = 5.80, p = 0.003). SOM ratio was not significant between groups (F [2;132] = 0.55, p = 0.576). Bonferroni multiple comparison tests revealed that individuals with manifest HD had significantly lower scores on all SOT conditions, composite score, falls, VIS, VEST and PREF sensory ratios than healthy controls and individuals with premanifest HD. There were no significant differences between healthy controls and individuals with premanifest HD (Table 2).

### LOS test

The analysis using the average scores revealed significant differences in LOS for endpoint excursion (F [2;107] = 13.34, p < 0.001), maximum excursion (F [2;107] = 14.60, p < 0.001), directional control (F [2;107] = 53.67, p < 0.001) and movement velocity (F [2;107] = 3.04, p = 0.052). However, no significant differences were evident between groups for reaction time (F [2;107] = 1.21, p = 0.302). Bonferroni multiple comparison test revealed that individuals with premanifest and manifest HD had significantly lower endpoint excursion (p < 0.001), maximum excursion (p < 0.002) and directional control (p < 0.006) values than healthy controls (Table 2). Maximum excursion and directional control were significantly lower (p = 0.024) in individuals with manifest HD compared to premanifest HD (Table 2). Movement velocity was slower, but did not reach significance in manifest HD compared to healthy controls (p = 0.062), and in manifest HD compared to premanifest HD (p = 0.083; Table 2). The analysis using specific directions revealed impairments towards RT, BWLT, FWLT and LT in premanifest HD compared to healthy controls on all LOS variables. Individuals with manifest HD had poorer postural stability towards all directions for MV, EE, ME and DC in comparison to healthy controls. Manifest HD individuals had postural stability deficits leaning towards all directions except LT on ME and DC variables when compared to individuals with premanifest HD (Supplementary Table 3).

### Association between postural stability tests, UHDRS-TMS, disease burden score CAG repeat and diagnostic confidence level

Spearman correlation coefficients revealed significant but low correlations between UHDRS-TMS, disease burden score, diagnostic confidence level outcomes, SOT and LOS outcomes within each group (Supplementary Fig. 1).

## Discussion

This study examined the validity of static and dynamic postural stability in a large cohort of premanifest HD, manifest HD and healthy age- and gender-matched individuals using computerised dynamic posturography. Significant impairments in static and dynamic postural stability and a greater number of falls were detected in individuals with premanifest and manifest HD compared to healthy age- and gender-matched controls. Significant but low associations were observed between impairments in static and dynamic postural stability and falls and clinical disease outcomes. Dynamic and static posturography, as measured by the SOT, demonstrated low to moderate reliability for all study groups.

Previous work by our team has shown that individuals with manifest HD, but not premanifest HD, have significant impairments in postural control at their limits of stability^4,12^. Our results partially align with these earlier findings. Here we found that when compared to healthy controls, individuals with both premanifest and manifest HD displayed significant impairments in postural control at the limits of stability, particularly distance and accuracy of movement. Discrepancies between the results could be attributed to smaller sample sizes used in previous studies that were therefore limited in power. An analysis of the direction of impairments revealed that individuals with premanifest HD have greater difficulty maintaining postural control when leaning in LT, BWLT and FWLT directions, while individuals with manifest HD experience greater difficulty maintaining postural control while leaning in FW, FWRT, BWLT, LT and FWLT directions. These results suggest that impairments in postural control arise on the left side of the body and extend to the front and right sides of the body as the disease progresses. The exact reason for early direction-specific impairments in postural stability is unknown. It is possible that lateralization of motor or sensory abnormalities occur as a result of contralateral brain dominancy in HD. This tentative supposition is supported by previous studies, which found that neuropathology may originate in the dominant left hemisphere and thus affect the right side of the body^18,19^. In our study, most of the participants in all groups were right handed, suggesting that postural abnormalities did not follow the same brain dominancy pattern. Although handedness is associated with contralateral brain dominancy, it is not yet known if this holds true for postural stability. Additional studies are required to investigate whether hemispheric changes in the brain are responsible for the observed direction-specific impairments.

Similar to previous findings^4,8,12^, we also found that individuals with manifest HD have significant impairments in postural control and a greater number of falls compared to healthy and premanifest HD individuals when sensory information is systematically manipulated. Impairments in postural control were particularly pronounced on conditions 5 and 6 of the SOT. During these conditions, individuals are provided with degraded somatosensory and visual information and must rely on vestibular information to maintain postural control. An analysis of sensory ratio scores revealed that individuals with manifest HD have greatest difficulty maintaining postural control when reliant only on vestibular information, indicating the presence of vestibular pathology. However, none of the assessed manifest HD individuals reported vestibular impairments and recent evidence shows that the vestibular system is preserved in individuals with mild to advanced HD^20^. It is therefore unlikely that vestibular pathology solely underpins impairments on the SOT in individuals with manifest HD. Contrary to previous findings^4^, individuals with premanifest HD, even those close to onset, did not display impairments in postural control on any condition of the SOT. The larger sample of individuals with HD within the present study likely accounts for these discrepancies in findings. An analysis of test-retest reliability revealed that the SOT measure had low to moderate reliability and differed depending on the condition and group. Manifest and premanifest HD participants had a more reliable performance in comparison with healthy controls. Studies examining the reliability of dynamic posturography in individuals with neurodegenerative disease is scarce, however studies using the SOT in healthy individuals have reported a wide range of ICC intervals, which is consistent with the values obtained by healthy controls in this study, and suggests a learning effect that it is more evident in healthy controls than in premanifest and manifest HD^21,22^.

Significant but low associations were observed between impairments in postural control and clinical measures of disease severity (UHDRS-TMS and DCL) and burden (DBS, CAP score and CAG repeat length). These findings suggest that genetic burden alone is not the sole factor underpinning postural control impairments in HD. Previous findings by our group found strong associations between postural stability outcomes and measures of cognitive function^23^. Similar findings have also been found in individuals with Parkinson’s disease^24^. This finding has important clinical implications. In particular, it suggests that cognitive enhancement therapies should be incorporated into rehabilitation interventions aimed at improving postural stability in individuals with HD. Additional longitudinal studies are needed to evaluate potential associations between postural stability and cognition in individuals with HD over time.

To date no studies have investigated the neural basis of static and dynamic postural control impairments in individuals with HD. However, degeneration within the striatum and brain stem of individuals with HD is believed to underpin postural control impairments^25^. The striatum and brain stem, along with other basal ganglia and cortical structures are crucially involved in sensorimotor integration, which is essential for maintaining normal postural control^26–30^. Studies in individuals with lesions in basal ganglia structures, such as Parkinson’s disease, have reported significantly poorer postural control^31,32^. Poorer postural control has also been reported in individuals with brain stem atrophy, such as patients with spinocerebellar ataxias and traumatic brain injuries^33–36^. Future studies are required to explore the neural basis of postural control impairments in individuals with HD.

This study is not without limitations. First this was a cross-sectional study, which does not provide information on changes in postural stability over time. Second, reliability analyses were only performed for the SOT. Future studies should continue investigating the reliability of the SOT and LOS test in HD, as an improved knowledge of the test-retest reliability of dynamic postural stability tests will improve their applicability for disease modifying drug trials. Third, individuals within the study continued their normal medication regimens, which may have improved or reduced performance on postural stability outcomes. Indeed, one patient was taking Tetrabenazine during testing procedures, which has been shown to improve performance on SOT outcomes previously^37^. Future studies should take into account medication as a variable affecting postural stability in HD.

Our findings show that impairments in static and dynamic postural control are common in individuals with premanifest and manifest HD and worsen with advancing disease. In addition, our findings show, for the first time, that impairments in postural stability commence on the left side of the body in individuals with premanifest HD. Although there is still a need to determine the sensitivity of these measures to postural changes over time; these findings provide compelling support for utilizing computerized dynamic posturography outcomes as a marker suitable for tracking the progression of HD, and potentially to determine the efficacy of upcoming disease modifying drug trials.

## Electronic supplementary material

Supplementary Information

## Data Availability

The dataset analysed during the current study is available from the main author upon reasonable request and with permission of the corresponding author.
