# Supplementary material for: Computerised Dynamic Posturography in Premanifest and Manifest individuals with Huntington’s Disease
Source: Sci Rep. 2018 Oct 2;8:14615. doi: 10.1038/s41598-018-32924-y (PMC6168504; doi:10.1038/s41598-018-32924-y)
Supplement: Supplementary file 1 — Supplementary Information [file 41598_2018_32924_MOESM1_ESM.pdf]

Computerised Dynamic Posturography in Premanifest and Manifest individuals with Huntington's Disease

Alvaro Reyes, PhD,[1] Danielle Salomonczyk, PhD,[2] Wei-Peng Teo, PhD,[3] Luis D. Medina, PhD,[4] Danielle Bartlett, MSc,[5] Eva Pirogovsky-Turk, PhD,[6] Pauline Zaenker, MSc,[5] Jody Corey Bloom, MD, PhD,[7] Roger Simmons, PhD,[8] Mel Ziman, PhD,[5,9] Paul Gilbert, PhD,[10] and Travis Cruickshank, PhD.[5,11,*]

[1]Facultad de Ciencias de la Rehabilitacion. Universidad Andres Bello, Santiago, Chile

[2]Department of Psychology, San Diego State University, San Diego, California, USA

[3]Institute for Physical Activity and Nutrition (IPAN), Deakin University

[4] Department of Psychology, University of Houston, Houston, Texas, USA

[5]School of Medical and Health Sciences, Edith Cowan University, Perth, Australia

[6]Department of Psychiatry, University of California San Diego, La Jolla, California, USA

[7]Department of Neurosciences, University of California San Diego, La Jolla, California

[8]School of Exercise and Nutritional Sciences, San Diego State University, San Diego, California, USA

[9]School of Biomedical Science, University Western Australia, Perth, Australia

[10]San Diego State University/University of California San Diego Joint Doctoral Program in Clinical Psychology, San Diego/La Jolla, California, USA

[11]Perron Institute for Neurological and Translational Science, Perth, Australia

***Corresponding Author:** t.cruickshank@ecu.edu.au

Supplementary Table 1. Number of participants at each study site

Study Site	Healthy control	Pre-manifest	Manifest
Melbourne	0	13	0
Perth	28	20	21
San Diego	17	22	11
Total	45	55	32

Supplementary Table 2. Intraclass correlation coefficient (ICC) and [95% confidence interval] for the SOT test.

	healthy control ICC [95% CI]	premanifest HD ICC [95% CI]	manifest HD ICC [95% CI]
Condition1	0.62 [0.47; 0.75]	0.42 [0.26; 0.58]	0.78 [0.66;0,87]
Condition2	0.42 [0.24; 0.59]	0.61 [0.47; 0.73]	0.72 [0.58; 0.84]
Condition3	0.49 [0.31; 0.65]	0.63 [0.49; 0.75]	0.56 [0.36; 0.73]
Condition4	0.60 [0.44; 0.74]	0.63 [0.50; 0.75]	0.70 [0.54; 0.82]
Condition5	0.27 [0.09; 0.47]	0.76 [0.66; 0.84]	0.69 [0.53; 0.82]
Condition6	0.47 [0.29; 0.63]	0.75 [0.64;0.84]	0.66 [0.48; 0.80]

SOT: sensory organization test.

Supplementary Table 3. Mean (standard deviation), and p-values of limits of stability test by specific directions for all groups

Postural Stability Outcomes		Healthy (n=45)	Premanifest HD (n=55)	Manifest HD (n=32)	p values		
					Healthy versus premanifest HD	Healthy versus manifest HD	Premanifest versus manifest HD
Reaction Time	Forward	1.15 (0.52)	1.08 (0.52)	0.93 (0.67)	0.831	0.491	0.708
	Right forward	0.87 (0.39)	0.79 (0.34)	0.92 (0.38)	0.554	0.911	0.524
	Right	0.85 (0.41)	0.87 (0.40)	0.87 (0.35)	0.983	0.990	1.000
	Right back	0.90 (0.39)	0.80 (0.39)	0.62 (0.34)	0.527	0.090	0.320
	Back	2.85 (14.07)	0.77 (0.49)	0.76 (0.62)	0.534	0.762	1.000
	Left back	0.87 (0.42)	0.62 (0.38)	0.90 (0.43)	0.013	0.972	0.087
	Left	0.72 (0.34)	0.68 (0.38)	0.68 (0.31)	0.866	0.940	1.000
	Left Forward	0.88 (0.34)	0.73 (0.43)	0.94 (0.55)	0.214	0.921	0.291
Movement velocity	Forward	2.86 (1.62)	3.48 (1.87)	3.34 (1.36)	0.221	0.704	0.965
	Right forward	2.86 (1.62)	3.48 (1.87)	3.34 (1.36)	0.221	0.704	0.965
	Right	4.16 (1.85)	4.43 (2.48)	4.92 (1.23)	0.828	0.557	0.776
	Right back	3.59 (1.63)	3.96 (1.72)	5.16 (2.62)	0.600	0.026	0.106
	Back	2.86 (1.38)	3.53 (1.75)	4.86 (4.04)	0.282	0.010	0.113
	Left back	3.95 (1.94)	3.57 (1.83)	4.72 (2.12)	0.626	0.447	0.156
	Left	4.43 (2.17)	4.22 (2.70)	5.76 (3.51)	0.924	0.277	0.168
	Left Forward	4.67 (2.23)	3.44 (1.85)	4.28 (2.10)	0.015	0.833	0.414
Endpoint excursion	Forward	63.81 (22.69)	57.69 (23.12)	34.41 (11.16)	0.403	<0.001	0.005
	Right forward	80. 04(18.34)	75.41 (23.35)	61.25 (21.72)	0.573	0.029	0.120
	Right	76.88 (14.08)	65.83 (20.72)	65.75 (14.31)	0.012	0.162	1.000
	Right back	69.02 (21.69)	65.15 (20.93)	62.15 (23.14)	0.678	0.601	0.904
	Back	49.11(14.74)	53.31 (19.99)	43.76 (22.97)	0.549	0.659	0.255
	Left back	71.79 (20.86)	53.79 (23.63)	44.46 (29.55)	0.001	0.002	0.437
	Left	75.5 (18.09)	58.64 (24.68)	58.38 (23.09)	0.001	0.053	0.999
	Left Forward	86.45 (17.52)	61.71 (28.61)	59.53 (23.75)	<0.001	0.003	0.959
Maximum excursion	Forward	86.81 (14.67)	82.16 (15.67)	61.16 (20.59)	0.366	<0.001	<0.001
	Right forward	95.41 (10.08)	96.60 (12.09)	77.41 (24.61)	0.910	<0.001	<0.001
	Right	89.02 (8.48)	86.49 (12.87)	83.33 (16.31)	0.579	0.338	0.704
	Right back	89.86 (14.16)	86.84 (15.59)	76.92 (22.41)	0.653	0.041	0.138
	Back	73.88 (13.40)	74.41 (22.35)	66.15 (27.19)	0.992	0.475	0.415
	Left back	91.62 (14.59)	76.20 (27.44)	70.30 (23.24)	0.005	0.015	0.704
	Left	89.56 (8.10)	73.13 (23.60)	71.23 (16.06)	<0.001	0.007	0.944
	Left Forward	97.75 (10.69)	77.90 (27.27)	76.46 (27.37)	<0.001	0.012	0.978
Directional control	Forward	89.20 (6.86)	84.32 (11.78)	47.83 (28.86)	0.203	<0.001	<0.001
	Right forward	83.69 (7.44)	81.73 (14.11)	52.58 (32.15)	0.820	<0.001	<0.001
	Right	84.60 (8.14)	80.92 (8.67)	65.91 (14.53)	0.165	<0.001	<0.001
	Right back	67.61 (15.33)	65.86 (19.97)	29.00 (29.62)	0.910	<0.001	<0.001
	Back	72.93 (19.56)	61.68 (26.24)	21.46 (26.90)	0.080	<0.001	<0.001
	Left back	66.81 (15.29)	58.75 (26.02)	30.30 (22.68)	0.208	<0.001	<0.001
	Left	84.72 (7.29)	70.77 (23.81)	59.76 (22.68)	0.002	<0.001	0.173
	Left Forward	84.90 (6.24)	71.25 (26.32)	48.53 (27.08)	0.007	<0.001	0.003

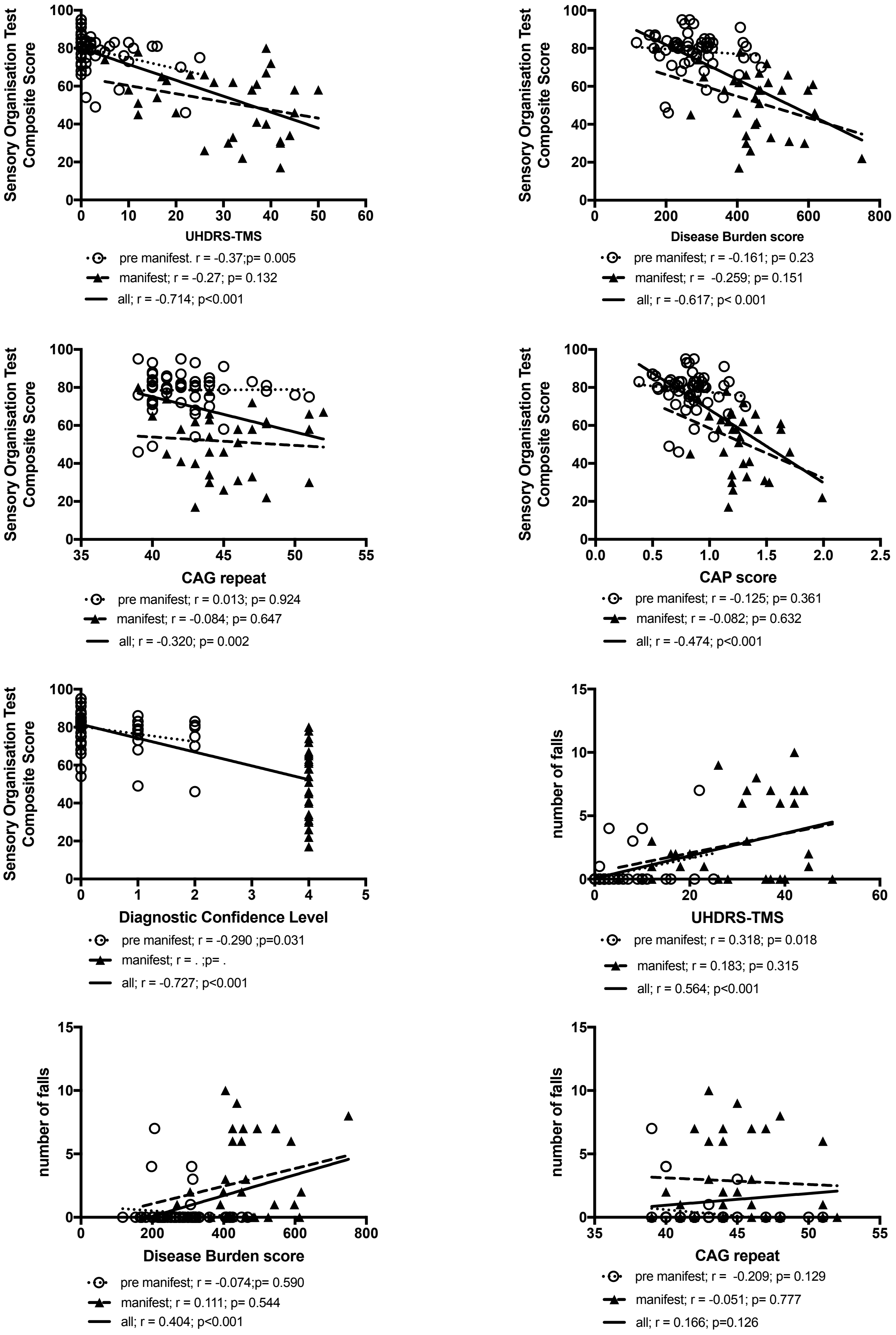
Sensory Organisation Test Composite Score
UHDRS-TMS
pre manifest. r = -0.37;p= 0.005
manifest; r = -0.27; p= 0.132
all; r = -0.714; p<0.001
Sensory Organisation Test Composite Score
Disease Burden score
pre manifest; r = -0.161; p= 0.23
manifest; r = -0.259; p= 0.151
all; r = -0.617; p< 0.001
Sensory Organisation Test Composite Score
CAG repeat
pre manifest; r = 0.013; p= 0.924
manifest; r = -0.084; p= 0.647
all; r = -0.320; p= 0.002
Sensory Organisation Test Composite Score
CAP score
pre manifest; r = -0.125; p= 0.361
manifest; r = -0.082; p= 0.632
all; r = -0.474; p<0.001
Sensory Organisation Test Composite Score
Diagnostic Confidence Level
pre manifest; r = -0.290 ;p=0.031
manifest; r = . ;p= .
all; r = -0.727; p<0.001
number of falls
UHDRS-TMS
pre manifest; r = 0.318; p= 0.018
manifest; r = 0.183; p= 0.315
all; r = 0.564; p<0.001
number of falls
Disease Burden score
pre manifest; r = -0.074;p= 0.590
manifest; r = 0.111; p= 0.544
all; r = 0.404; p<0.001
number of falls
CAG repeat
pre manifest; r = -0.209; p= 0.129
manifest; r = -0.051; p= 0.777
all; r = 0.166; p=0.126

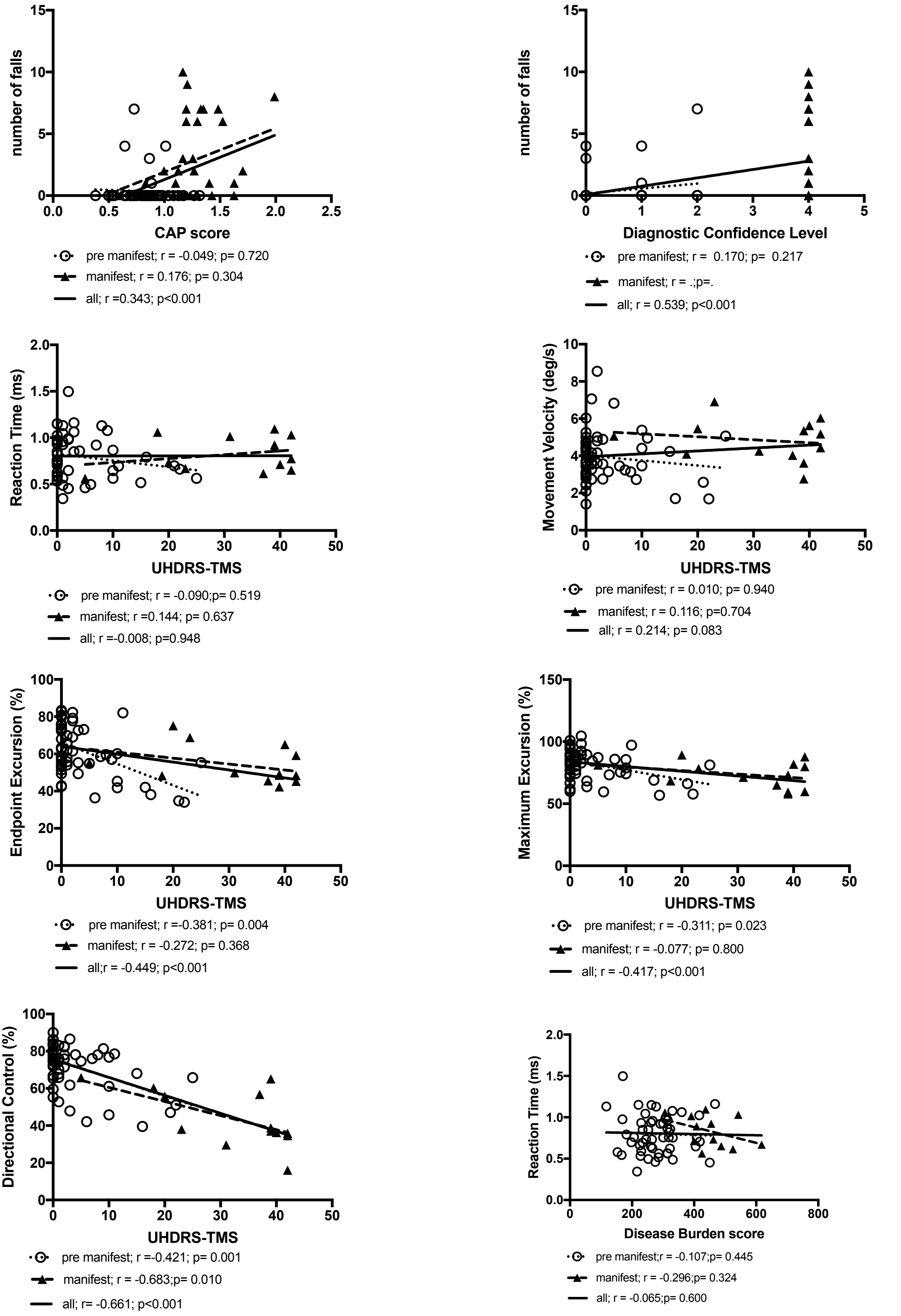

number of falls
CAP score
pre manifest; r = -0.049; p= 0.720
manifest; r = 0.176; p= 0.304
all; r =0.343; p<0.001
number of falls
Diagnostic Confidence Level
pre manifest; r = 0.170; p= 0.217
manifest; r = .;p=.
all; r = 0.539; p<0.001
Reaction Time (ms)
UHDRS-TMS
pre manifest; r = -0.090;p= 0.519
manifest; r =0.144; p= 0.637
all; r =-0.008; p=0.948
Movement Velocity (deg/s)
UHDRS-TMS
pre manifest; r = 0.010; p= 0.940
manifest; r = 0.116; p=0.704
all; r = 0.214; p= 0.083
Endpoint Excursion (%)
UHDRS-TMS
pre manifest; r =-0.381; p= 0.004
manifest; r = -0.272; p= 0.368
all;r = -0.449; p<0.001
Maximum Excursion (%)
UHDRS-TMS
pre manifest; r = -0.311; p= 0.023
manifest; r = -0.077; p= 0.800
all; r = -0.417; p<0.001
Directional Control (%)
UHDRS-TMS
pre manifest; r =-0.421; p= 0.001
manifest; r = -0.683;p= 0.010
all; r= -0.661; p<0.001
Reaction Time (ms)
Disease Burden score
pre manifest;r = -0.107;p= 0.445
manifest; r = -0.296;p= 0.324
all; r = -0.065;p= 0.600

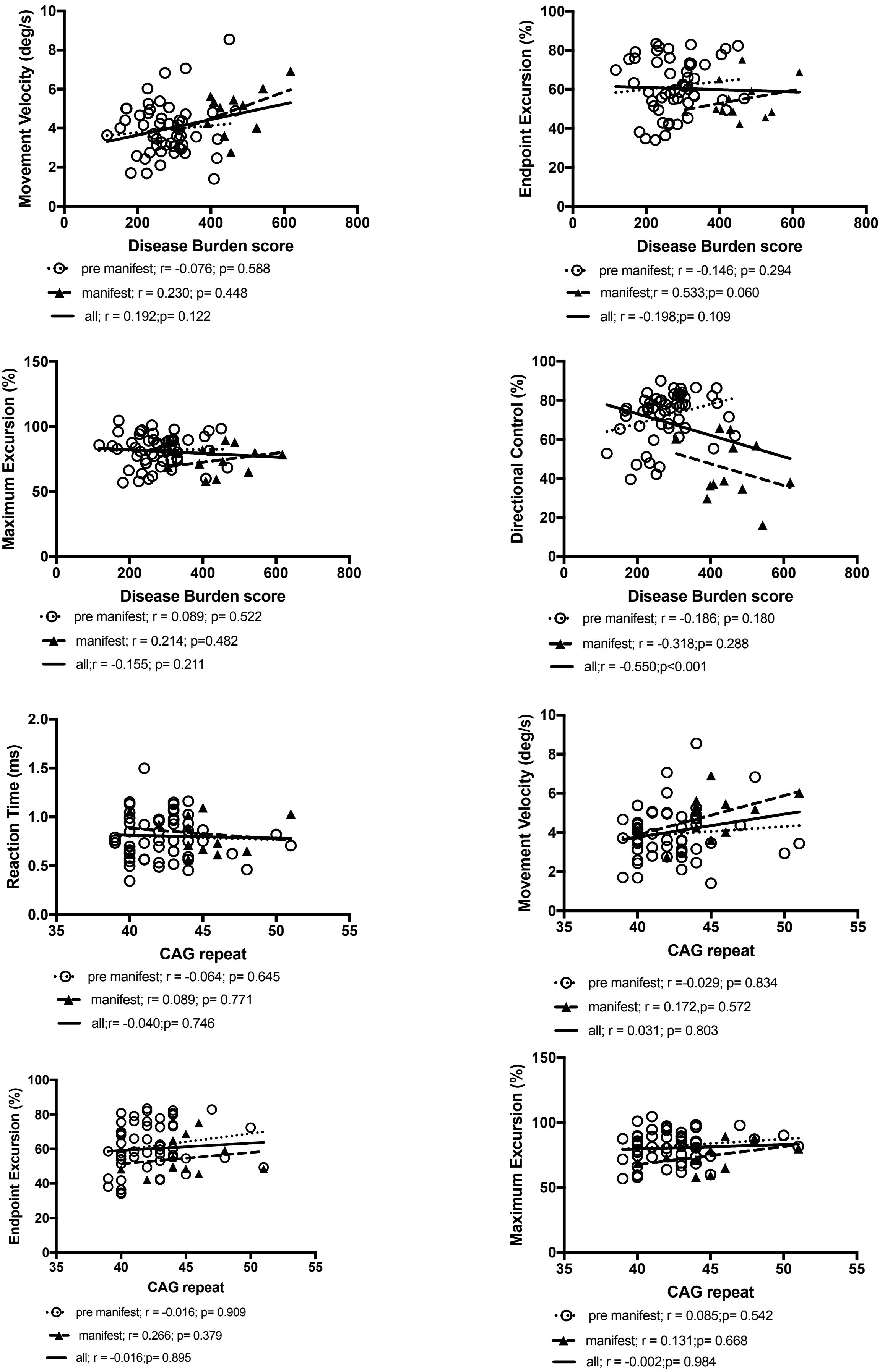
Movement Velocity (deg/s)
Disease Burden score
pre manifest; r= -0.076; p= 0.588
manifest; r = 0.230; p= 0.448
all; r = 0.192;p= 0.122
Endpoint Excursion (%)
Disease Burden score
pre manifest; r = -0.146; p= 0.294
manifest;r = 0.533;p= 0.060
all; r = -0.198;p= 0.109
Maximum Excursion (%)
Disease Burden score
pre manifest; r = 0.089; p= 0.522
manifest; r = 0.214; p=0.482
all;r = -0.155; p= 0.211
Directional Control (%)
Disease Burden score
pre manifest; r = -0.186; p= 0.180
manifest; r = -0.318;p= 0.288
all;r = -0.550;p<0.001
Reaction Time (ms)
CAG repeat
pre manifest; r = -0.064; p= 0.645
manifest; r= 0.089; p= 0.771
all;r= -0.040;p= 0.746
Movement Velocity (deg/s)
CAG repeat
pre manifest; r =-0.029; p= 0.834
manifest; r = 0.172,p= 0.572
all; r = 0.031; p= 0.803
Endpoint Excursion (%)
CAG repeat
pre manifest; r = -0.016; p= 0.909
manifest; r= 0.266; p= 0.379
all; r = -0.016;p= 0.895
Maximum Excursion (%)
CAG repeat
pre manifest; r = 0.085;p= 0.542
manifest; r = 0.131;p= 0.668
all; r = -0.002;p= 0.984

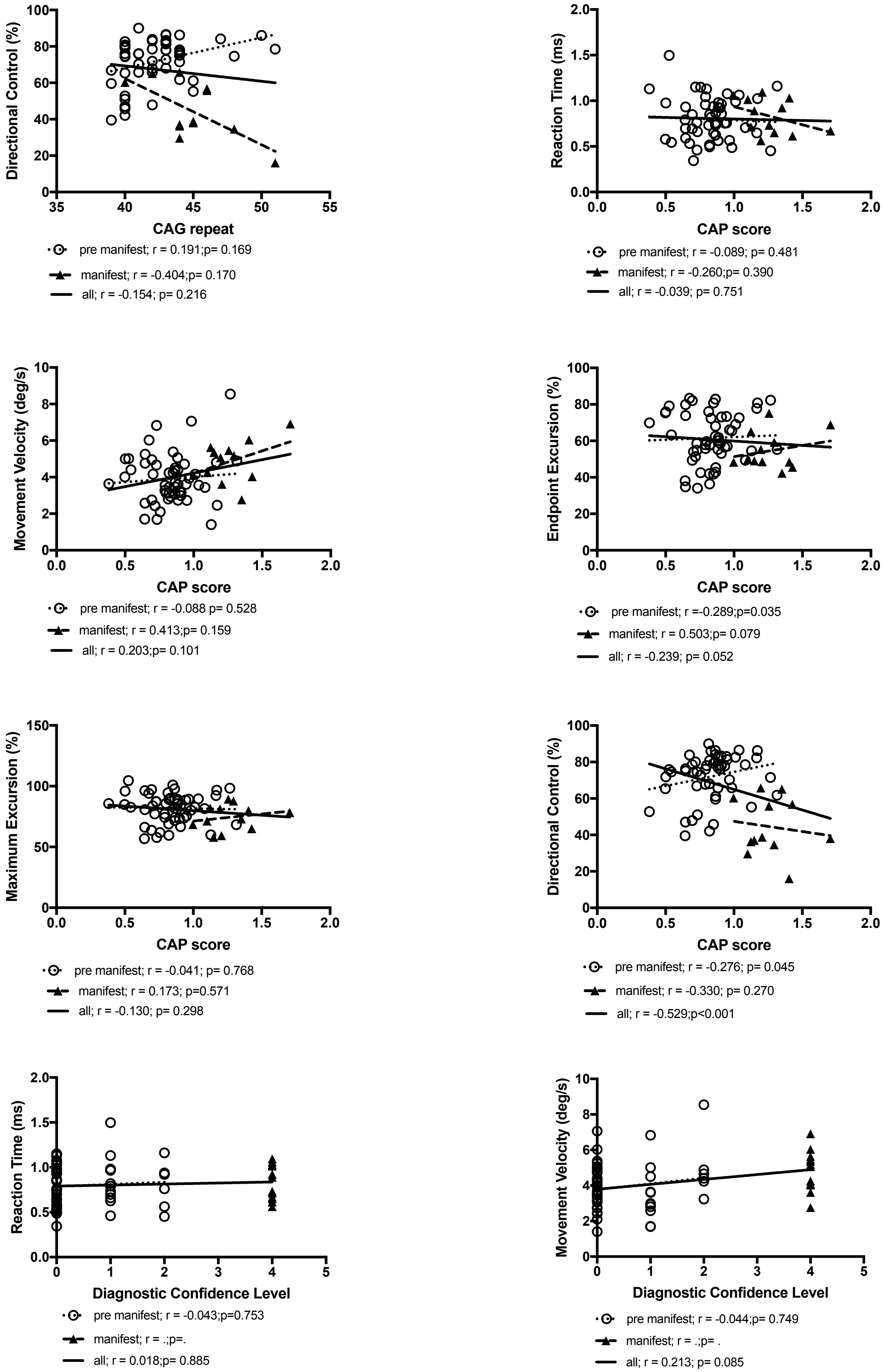
Directional Control (%)
CAG repeat
pre manifest; r = 0.191;p= 0.169
manifest; r = -0.404;p= 0.170
all; r = -0.154; p= 0.216
Reaction Time (ms)
CAP score
pre manifest; r = -0.089; p= 0.481
manifest; r = -0.260;p= 0.390
all; r = -0.039; p= 0.751
Movement Velocity (deg/s)
CAP score
pre manifest; r = -0.088 p= 0.528
manifest; r = 0.413;p= 0.159
all; r = 0.203;p= 0.101
Endpoint Excursion (%)
CAP score
pre manifest; r =-0.289;p=0.035
manifest; r = 0.503;p= 0.079
all; r = -0.239; p= 0.052
Maximum Excursion (%)
CAP score
pre manifest; r = -0.041; p= 0.768
manifest; r = 0.173; p=0.571
all; r = -0.130; p= 0.298
Directional Control (%)
CAP score
pre manifest; r = -0.276; p= 0.045
manifest; r = -0.330; p= 0.270
all; r = -0.529;p<0.001
Reaction Time (ms)
Diagnostic Confidence Level
pre manifest; r = -0.043;p=0.753
manifest; r = .;p=.
all; r = 0.018;p= 0.885
Movement Velocity (deg/s)
Diagnostic Confidence Level
pre manifest; r = -0.044;p= 0.749
manifest; r = .;p= .
all; r = 0.213; p= 0.085

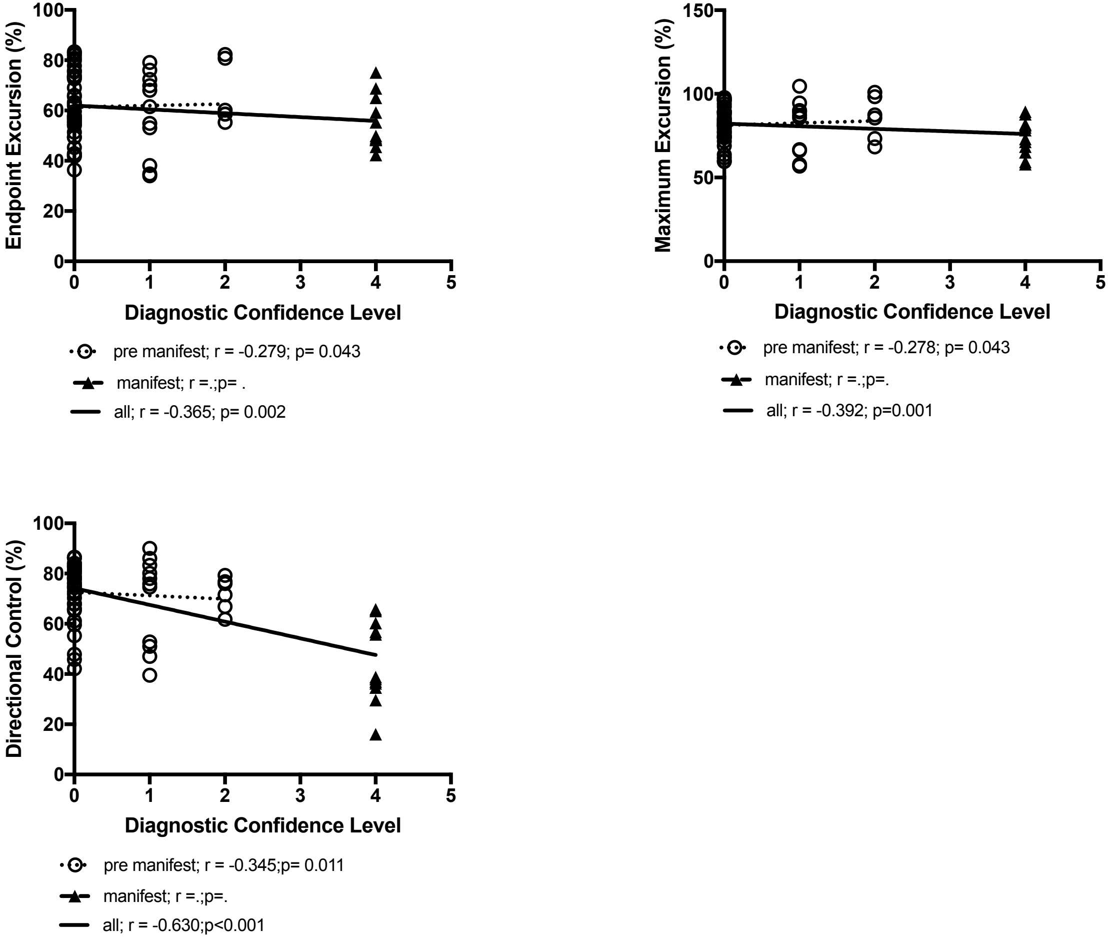

Supplementary Figure 1. Spearman correlation coefficient (r) and p-values (p) between dynamic posturography variables and UHDRS-TMS, disease burden score, CAG repeat, CAP score and diagnostic confidence level in premanifest (circles) and manifest (triangles) individuals with HD. Dashed lines show regression slopes for premanifest and manifest HD. Continuous line show regression slope for pooled groups.
